# Modification of the gate electrode by self-assembled monolayers in flexible electrolyte-gated organic field effect transistors: work function *vs.* capacitance effects†

**DOI:** 10.1039/c8ra05300f

**Published:** 2018-08-02

**Authors:** Francesca Leonardi, Adrián Tamayo, Stefano Casalini, Marta Mas-Torrent

**Affiliations:** Institut de Ciència de Materials de Barcelona (ICMAB-CSIC), Networking Research Center on Bioengineering, Biomaterials and Nanomedicine (CIBER-BBN), Campus de la Universitat Autònoma de Barcelona Cerdanyola E-08193 Barcelona Spain mmas@icmab.es

## Abstract

Understanding the physics behind the operational mechanism of Electrolyte-Gated Organic Field-Effect Transistors (EGOFETs) is of paramount importance for the correct interpretation of the device response. Here, we report the systematic functionalization of the gate electrode of an EGOFET with self-assembled monolayers with a variety of dipolar moments showing that both the chemical nature and the monolayer density influence the electrical characteristics of the device.

## Abstract 2

The functionalisation of the gate electrode in electrolyte-gated field effect transistors (EGOFETs) with self-assembled monolayers effect the device electrical performance mainly due to the induced capacitance changes.

## Graphical abstract

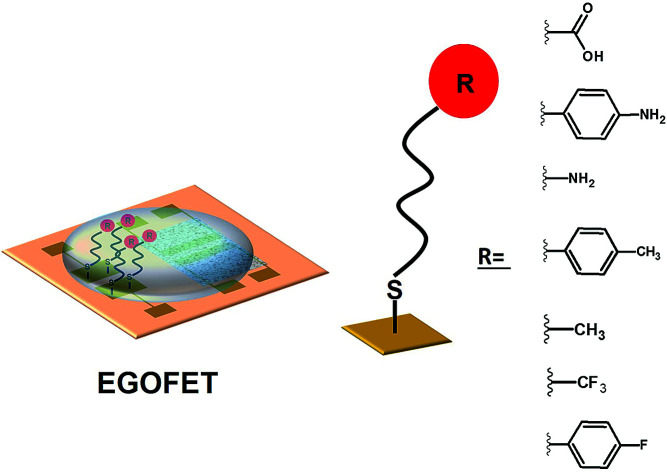

## Introduction

Nowadays, fabrication of organic electronic components is accomplished with low-cost and low-temperature methods over a large area and virtually on every substrate. However, the investigation of efficient fabrication processes accompanied by excellent output performances remains quite challenging. Device performance and life-time are not only dependent on active materials themselves, but the role of interfaces has been revealed to be pivotal for the realization of an efficient and robust electronic platform.

Organic Field-Effect Transistors (OFETs) are not an exception. In these devices, an organic semiconductor (OSC) deposited on a dielectric is brought into contact with two electrodes (*i.e*., source and drain). Then, by the application of an electric field along the dielectric (*i.e.*, gate-source voltage, *V*_GS_) combined with a longitudinal electric field (*i.e.* drain-source voltage, *V*_DS_) the conductivity along the first OSC molecular monolayers close to the dielectric is modulated. Hence, these devices are highly sensitive to both the OSC/electrode and OSC/dielectric interfaces.^1,2^ Consequently, different strategies have been devised to modify such interfaces in order to tune and optimize the final device performance. Among them, the surface modification by means of self-assembled monolayers (SAMs) represents a valuable and widely explored route in organic electronics due to its versatility and simplicity.^3,4^

SAMs have been grown on the oxide dielectric in OFETs mainly to reduce the charge trapping that is typically induced by the surface –OH groups,^5^ to control the OSC crystal growth,^6^ to tune the surface wettability,^7^ which is crucial for the deposition of OSCs from solution, to modify the density and nature of the charge carriers^8,9^ and to implement additional functionalities such as switchable systems.^10,11^ Many efforts have also been focused on the modification of the source/drain (S/D) electrodes. For instance, SAMs on the S/D can tune the electrode–OSC interaction leading to changes in the nucleation and growth of the OSC deposited on top.^12–16^ However, SAMs on the S/D have mainly been exploited to improve the device contact resistance. The metals functionalization using SAMs featuring different dipole moments impacts directly on their work function (*φ*_m_).^17^ In this way, a better alignment of *φ*_m_ with the organic semiconductor energy levels can be achieved ensuring a more efficient charge injection.^18^

The *φ*_m_ of the gate electrode has also a clear impact on the device characteristics since it determines the flat band potential, which is, in turn, related to the threshold voltage (*V*_th_). Hence, a linear dependence between *φ*_m_ and *V*_th_ has been previously observed in devices wherein different gate metals have been employed.^19^ However, due to configuration restrictions, the tuning of the gate electrode work function employing SAMs is not viable in conventional OFETs. On the contrary, Electrolyte-Gated Organic Field-Effect Transistors (EGOFETs) open the way towards the gate functionalization, as already demonstrated for the development of biosensors.^26–28^ These electronic devices employ an electrolyte media as dielectric, where the gate contact is immersed.^20,21^ The application of a *V*_GS_ leads to the formation of two electrical double layers (EDLs) at the OSC/electrolyte and electrolyte/gate interfaces, which determine the device capacitance. EGOFETs or water-gated FETs employing different gate metals have also been investigated, observing in most of the cases a shifting of the device *V*_th_ as a function of the metal *φ*_m_ but exhibiting clear deviations from linearity.^22–25^

Here, mimicking the commonly used strategy of modifying S/D electrodes deploying SAMs with a range of dipole moments to tune their work function, we have modified the Au gate electrode of flexible EGOFETs. A coplanar gate electrode has been employed which has been systematically modified by means of a series of thiolated SAMs bearing different terminal groups. Interestingly, and oppositely to what it was previously found in devices where the gate metal was modified, we did not observe a clear correlation between the modification of the metal work function with the SAMs and the device performance, but instead, the changes in capacitance played a crucial role..

## Experimental

### Methods

Dibenzotetrathiafulvalene (DBTTF, purity 97%) and polystyrene (PS, MW ≈ 10 000 g mol^−1^) were purchased from Sigma-Aldrich and used without further purifications. 4 wt% DBTTF and PS solutions were prepared in chlorobenzene (CB) in a volume ratio of 1 : 2 DBTTF : PS. 1*H*,1*H*,2*H*,2*H*-Perfluorodecanethiol (PFDT), 1-dodecanethiol (1DT), 4-methylthiophenol (MeTP), 4-aminothiophenol (ATP), 16-mercaptohexadecanoic acid (MHD), 4-fluorothiophenol (FTP) and 11-amino-1-undecanol (AUT) were purchased from Sigma-Aldrich and used without further purification. All SAMs were prepared from a 1 mM solution in ethanol.

Dextran (from *Leuconostoc mesenteroides* MW = 64.000–76.000) was purchased from Sigma-Aldrich and used without further treatments. Kapton® foils were purchased from DuPont (Kapton®HN, 75 μm thick) and cleaned with acetone and isopropanol before use.

### EGOFET fabrication and SAM printing

Source, drain and gate electrodes were patterned on Kapton® foil (75 μm thick, DuPont) using positive photolithography and a thin metal layer (5/40 nm, Cr/Au) was thermally evaporated (system Auto 306 from BOC Edward). The channel length and width were fixed to 30 μm and 19 680 μm, namely a *W*/*L* = 656. Before the deposition of the blend solutions, substrates were cleaned in ultrasonic bath with acetone and isopropanol, and then dried under nitrogen flow.

DBTTF : PS blend was coated on Kapton® substrates through Bar-Assisted Meniscus Shearing (BAMS) technique at a speed of 1 cm s^−1^ and keeping temperature plate equal to 105 °C. As confirmed by XRD analysis, the crystalline thin-film is dominated by DBTTF γ-phase (see Fig. S1†).^29,30^

Semiconductor patterning was achieved by first drop casting a dextran solution (10 mg ml^−1^ in water) on the gate region and letting it dry for 10 minutes. Afterwards, the DBTTF : PS blend thin film was deposited as previously mentioned. Subsequently, the device was gently rinsed with water to allow the complete removal of the dextran sacrificial layer, exposing hence the gate electrode and leaving unaltered the OSC thin-film.

Self-Assembled Monolayers (SAMs) were deposited onto gate electrode by means of a PDMS-assisted printing. A 5 × 5 mm^2^ PDMS stamp with 2 μl of solution containing the self-assembling molecules was printed on the gate electrode for 2 minutes and subsequently rinsed with MilliQ water. For the growth of the SAMs on gold disks and on the gold slides employed for contact angle measurements, a 1 mM solution in ethanol of the target molecule was prepared and the electrode (*Ø* = 1.6 mm) or the gold substrate (1 cm^2^) was immersed in it overnight.

### Characterization

Electrolyte-gated field-effect transistors were characterized by an Agilent B1500A semiconductor device analyzer under ambient conditions. All the measurements were performed in MilliQ (*σ* ≈ 18 MΩ) water.

*I*–*V* transfer characteristics (*I*_DS_*vs. V*_GS_) were recorded in linear regime keeping the drain voltage equal to −0.1 V and the transistor electrical parameters were extracted from the characteristic curves.

Electrochemical characterization was carried out with NOVO control equipped with a POT/GAL 30 V/2 A electrochemical interface. SAMs quality was evaluated through Electrochemical Impedance Spectroscopy (EIS) in a standard three-electrode configuration, where Pt and Ag/AgCl act as counter and reference electrodes, respectively, and the SAM coated-gold represents the working electrode.

AC amplitude was fixed to 5 mV and the corresponding DC voltage to 0.4 V in order to monitor the monolayer behavior in the ON state of the device, *i.e. V*_GS_ = 0.4 V.

Optical microscope images were taken using the Olympus BX51 equipped with polarizer and analyser.

X-ray diffraction measurements were carried out with a PANalytical X'Pert Pro MRD (Materials Research Diffractometer) diffractometer. The used Cu K-alpha radiation was 1.54187 Å.

Kelvin Probe Force Microscopy (KPFM) data were obtained with a 5500LS SPM from Agilent Technologies in amplitude mode by applying an AC voltage (*V*_AC_) plus a DC voltage (*V*_DC_) to the sample. All the measurements were recorded with a gold tip with except for the NH_2_-terminated SAMs where a Pt one was employed. The CPD values extracted for each SAM have been compared with a reference gold substrate.

EGOFET parameters have been extracted according to the equation in linear regime:1
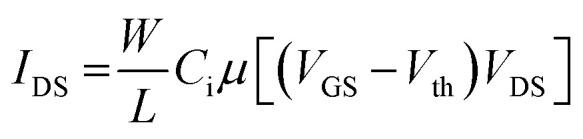
where *W* and *L* are the channel width and length, respectively, *C*_i_ the dielectric capacitance per unit area, *μ* the field-effect mobility, *V*_th_ the threshold voltage, *V*_GS_ the gate voltage, *V*_DS_ the drain voltage and *I*_DS_ the drain-source current.

Assuming a constant OSC mobility, the change in capacitance caused by the gate electrode modification has been estimated by evaluating the product of the device mobility by the dielectric capacitance before and after the functionalization:2
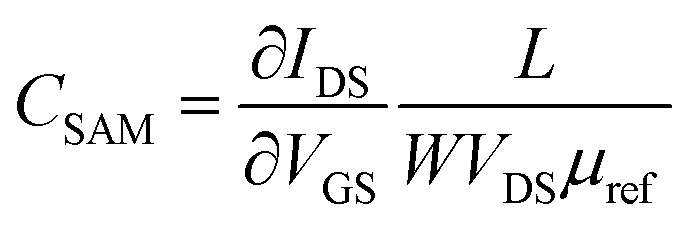
where *C*_SAM_ represents the dielectric capacitance obtained after SAM incorporation and *μ*_ref_ stands for the charge carrier mobility of the reference device (*i.e.*, with a bare gold gate electrode).^31^

## Results and discussion

### Device fabrication and EGOFET characterization

Aiming at flexible electronics applications, the selection of a flexible substrate together with a deposition technique compatible with roll-to-roll processes to deposit the OSC is pivotal. The widespread choice of Kapton®, as electronic substrate, resided in its good balance between physical and chemical properties as well as for its proved biocompatibility.^32^ Further, we selected to use as active material a blend of DBTTF with PS (*i.e.*, DBTTF : PS) deposited by the Bar-Assisted Meniscus Shearing (BAMS) technique following the previously reported conditions.^13,29^ It has been shown that this methodology can produce highly crystalline films with high reproducibility, which can be applied as active materials in OFETs as well as in EGOFETs exhibiting excellent performances.^29,31,33^

**Fig. 1:**
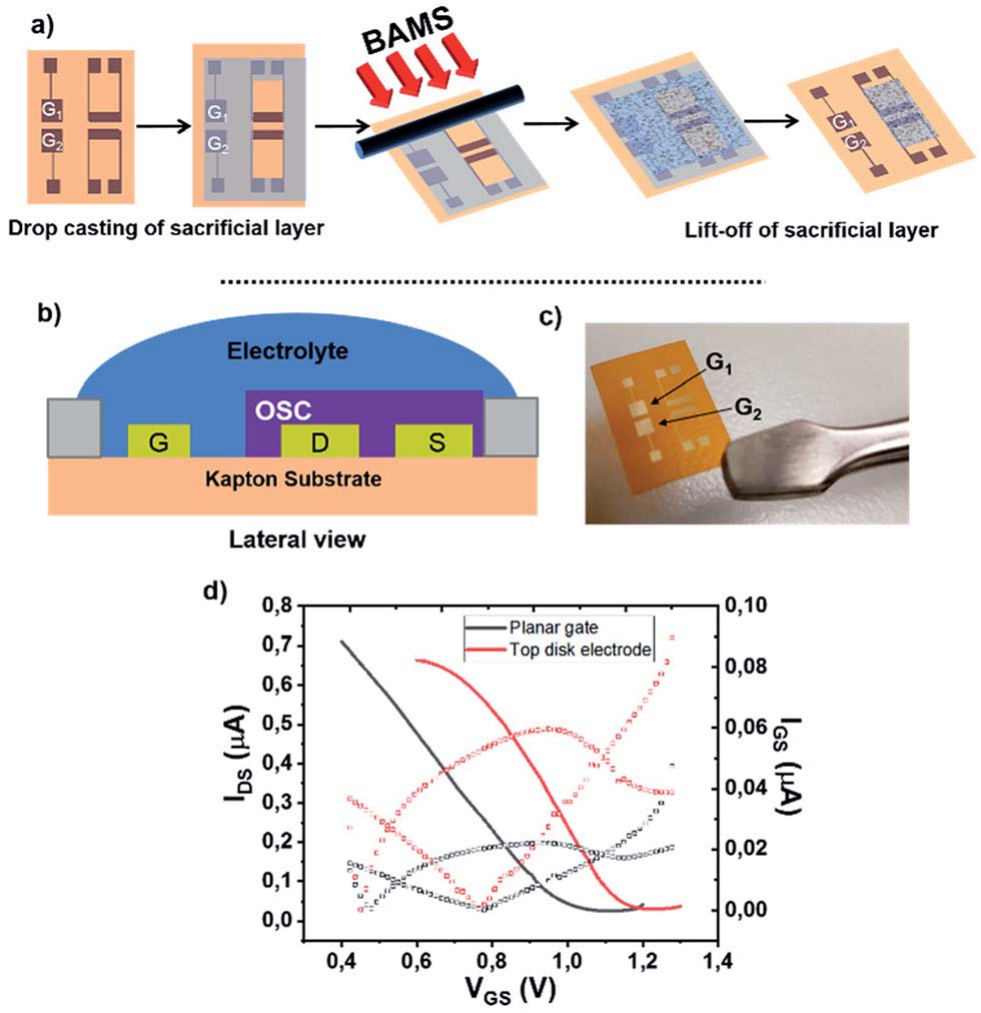
(a) Schematic representation of the semiconductor patterning showing two devices composed of two interdigitated source/drain electrode structures with their corresponding coplanar gate electrodes (G_1_ and G_2_). First, the dextran is casted, then the DBTTF : PS blend is deposited by BAMS and, finally, dextran is lifted of. (b) Lateral view of one EGOFET structure showing the co-planar gate electrode and a PDMS pool where water is deposited. (c) A photograph of the final device. (d) Electrical comparison of the device with a coplanar gate electrode with one with a standard top gate Au disk (linear regime *V*_DS_ = −0.1 V).

Fig. 1 shows the EGOFET configuration. Prior to the OSC deposition, the S/D electrodes were photolithographically patterned as well as the coplanar gate electrode placed 2 mm far from the channel area in order to facilitate the gate functionalization. This distance is appropriate considering the fast cations/anions diffusion.^34^ Aiming to avoid polarization drawbacks, the gate area is the double of the conductive channel, namely 0.02 cm^2^.

As previously mentioned, the coplanar configuration demands for the selective deposition of our semiconducting blend solely onto source and drain electrodes, thereby keeping clear the gate one. In order to do that, we made use here of sacrificial layers. These polymeric coatings offer many advantages such as low-cost processing and an extreme versatility of deposition.^35^ Among these sacrificial layers, the sub-class of water-soluble polymers have been already used for micromachining processes,^36^ as nano-adhesive plaster^37^ and in thin-film transistor technology.^38^ In our particular case, a dextran-based coating was employed to protect efficiently the gate electrode during DBTTF : PS deposition. Afterwards, the polymer was lifted-off in bi-distilled water. This way of patterning is extremely appealing, because it gets rid of organic solvents, which can easily damage the OSC thin-film. The whole process is schematized in Fig. 1a and the final device architecture is shown in Fig. 1b and c.

The proper patterning of the DBTTF-based thin-film has been easily verified by optical microscope, whose images show the gate electrode free of semiconductor, as displayed in Fig. S2b (ESI†). The DBTTF-based coating shows high homogeneity and crystallinity with plate-like crystalline domains. The thin film on Kapton® resembles the thin film structure obtained on silicon oxide substrates, where the γ polymorph of DBTTF is present.^30^ As a result, the XRD peak centered at 6.55° is the typical fingerprint for γ-phase (Fig. S1 ESI†), which is kinetically favored compared to the α-phase.^30^ Furthermore, the whole manufacturing has negligible effects on gold features, in fact root mean square roughness, *σ*_rms_, is comparable with untreated gold electrode (Fig. S2c and e†). Moreover, thin film coating seems to smooth source and drain surfaces and the organic coating appears as a compact thin layer. An opposite scenario takes place in the channel region, where *σ*_rms_ raises up after the thin film deposition (Fig. S2e and f†).

The electrical performances of the coplanar configuration have been compared with the staggered one using a top Au disk placed in the electrolytic medium. As depicted in Fig. 1d, the off current as well as the leakage one (*I*_GS_) is lower in the coplanar configuration with respect to the top gate. The two electrical performances are very similar, even though a slight less positive switch on voltage is observed in the coplanar configuration. These slight differences are ascribed to the different polycrystalline Au surfaces and to geometrical discrepancies between the coplanar and staggered configurations. The transfer and output characteristic recorded in saturation regime (*V*_DS_ = −0.4 V) are reported in Fig. S3.† Furthermore, a shelf-stability test, reported in Fig. S4,† demonstrated the robustness of the EGOFET even after one month. Despite of the weak amplification (namely ON/OFF ratio around 2), these devices show good mobility of the order 0.03 cm^2^ V^−1^ s^−1^, with an average value settled just one order of magnitude lower than its bottom gate/bottom contact counterpart.

### Gate electrode engineering *via* self-assembled monolayers

**Fig. 2:**
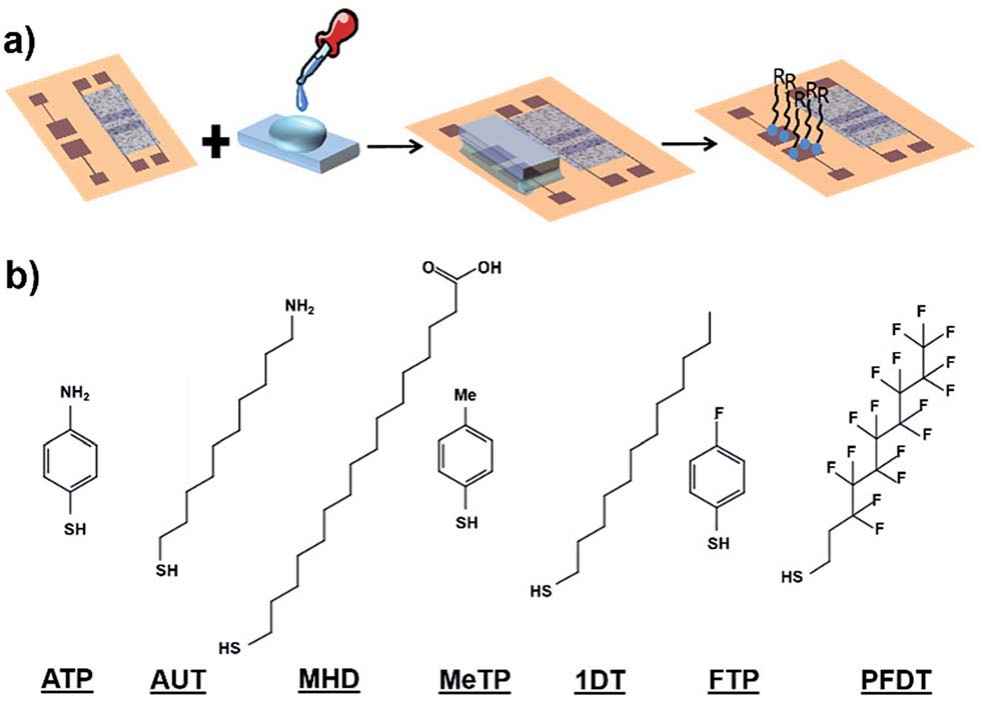
(a) Schematic diagram of PDMS-assisted printing of self-assembled monolayers. (b) Molecular structure of the SAMs subject of this study.

SAMs of 2*H*,2*H*-perfluorodecanethiol (PFDT), 1-dodecanethiol (1DT), 4-methylthiophenol (MeTP), 4-aminothiophenol (ATP), 16-mercaptohexadecanoic acid (MHD), 4-fluorothiophenol (FTP) and 11-amino-1-undecanol (AUT) have been used to modify the gate properties (Fig. 2b). All these molecules differ in their backbone length and their dipole moment which is determined by their functional groups.

Concerning the functionalization of the coplanar gate, the geometrical constrains of the chip along with the harmful effect of organic solvent in direct contact with the organic semiconductor did not allow us to proceed with the conventional solution immersion protocol. Thus, as alternative approach, we employed the “PDMS-assisted printing” (see Fig. 2a and the Experimental section).

**Fig. 3:**
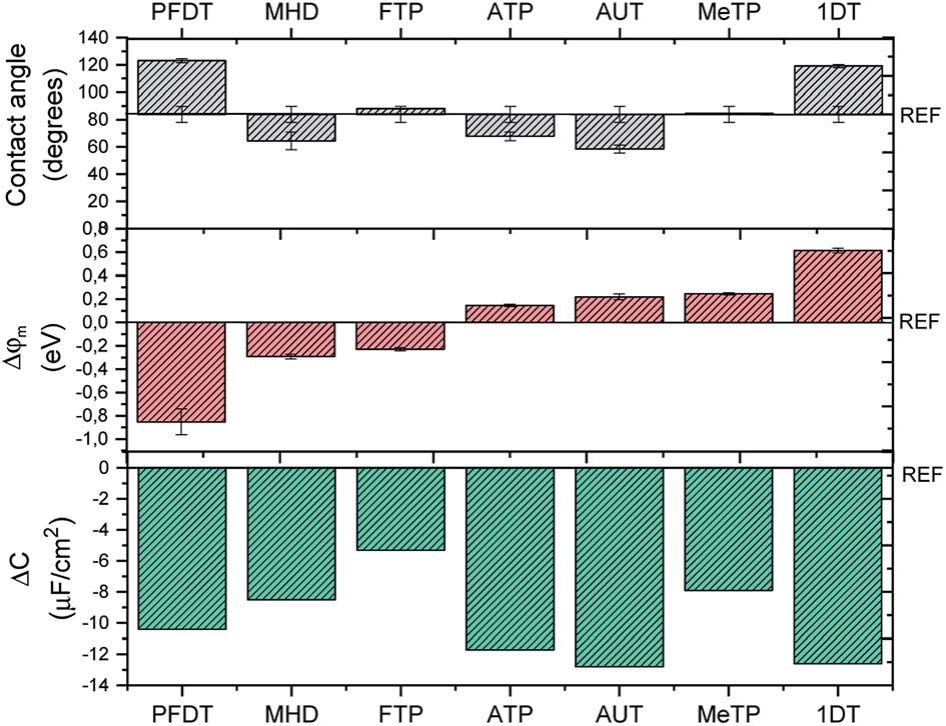
(a) Values of contact angle (*θ*_w_) measured in water on SAMs-modified gold substrates. (b) Shift of the work function (*φ*_m_) with respect to bare gold (4.7 eV) measured by KPFM; each SAM-coated surface is prepared by means of PDMS-assisted printing. *φ*_m_ are calculated by comparing the CPD values extracted for each SAM with a reference gold substrate free of any coating. (c) Decrease of double-layer capacitance (Δ*C*) after SAMs coating by means of PDMS-assisted printing. The measurements have been performed by EIS and each gold surface acts as working electrode. The values have been compared to the capacitance of bare gold (17.2 μF cm^−2^).

Contact angle measurements are one of the most exploited techniques to distinguish the hydrophilic/hydrophobic character of a SAM coated surface. As depicted in Fig. 3a, the wettability behavior of the SAMs can be grouped into three regions: (i) more hydrophilic surfaces (*i.e.*, lower contact angle values) than the bare Au, namely the ones coated by AUT, MHD and ATP, (ii) surfaces comparable to the bare Au, such as the ones coated by MeTP and FTP that likely have a low surface organization and packing and (iii) more hydrophobic surfaces (*i.e.*, higher contact angle values), namely the ones coated by 1DT and PFDT.

As mentioned, the interface dipole created by the SAM can cause an increase or decrease of the metal work function, which is directly related to the length of the backbone chain and to the nature of the terminal group. For example, Boudinet *et al.* reported that gold work function can be shifted with SAMs of ATP, MeTP and PFDT by 0.11, 0.47 and −0.69 eV, respectively.^39^ Here, we studied by Kelvin Probe Force Microscopy our series of SAMs printed on gold slides in order to estimate the modulation of the gold work function induced by the chemical modification. In Fig. 3b, *φ*_m_ shifts are reported for each SAM. As expected, according to the chemical nature of each molecule, *i.e.* the electron withdrawing or electron donating character of the SAM's head group, we observed a positive *φ*_m_ shift with 1DT, MeTP, AUT and ATP, whilst the opposite tendency was found with the SAMs of FTP and PFDT. Carboxylic-terminated SAMs represent a particular class, because the interface dipole can be modulated according to the length of the backbone chain.^40^ In the present case, MHD is long enough to behave similarly to a normal alkanethiol. However, KPFM data show a response similar to FTP and PFDT, which could be due to a poor monolayer ordering. It should be noted that these measurements are performed in air and it is well-known that the metal work function is altered in water environment.^20,41^

The SAM-modified surfaces were also characterized by means of electrochemical impedance spectroscopy (EIS) (Fig. S5†). From these data, it is possible to extract the double layer capacitance (*C*_dl_). As previously reported,^42^*C*_dl_ diminishes with respect to bare gold (17.2 μF cm^−2^) for each SAM (Fig. 3c), proving that our functionalization protocol is effective. As expected, the *C*_dl_ decrease is more pronounced in the case of the longest and more compact SAMs, namely AUT, 1DT, PFDT.

### EGOFET characterization

The following step was the electrical characterization of the EGOFETs whose gate had been previously functionalized with a SAM (at least 4 devices for each SAM). For comparison, the devices have also been measured using a gold disk gate electrode since, in this case, the standard procedure for SAM preparation (*i.e*., immersion for 12 hours in a 1 mM ethanol solution) was possible to apply. In this way, the influence of the SAM printing technique can also be assessed. Each device was firstly measured with a bare gate electrode, afterwards the electrode was functionalized with a SAM and then the device characteristics were recorded again.

**Fig. 4:**
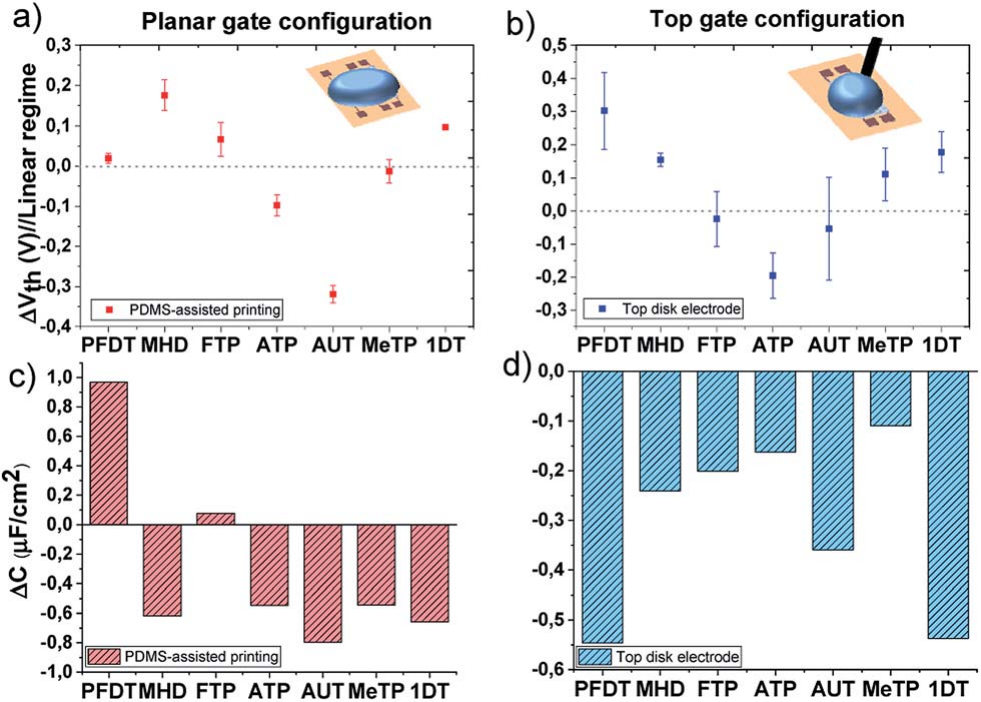
Threshold voltage shifts extracted from transfer characteristic in linear regime (*V*_DS_ = −0.1 V) and the corresponding capacitance shifts calculated according to eqn (2); The EGOFETs are measured by means of (a) and (c) the planar gate functionalized with SAMs by PDMS-assisted printing and (b) and (d) by using a gold disk electrode functionalized with a standard protocol.

The transfer characteristics measured for all the devices using different SAMs are shown in Fig. S6.† It is clearly observed that *V*_th_ changes are occurring for each SAM functionalization. The upper panels of Fig. 4 represent the threshold voltage shift (Δ*V*_th_ = *V*_th(SAM)_ − *V*_th(ref)_) of the functionalized devices with respect to the ones measured with the reference bare gold electrode. Notice that the SAMs in the plots are ordered from lower to higher work function. Comparing Fig. 4a and b, it can be noted that although the absolute values found for the two EGOFET configurations are different, the general Δ*V*_th_ trend is rather similar. These discrepancies can be ascribed to the different way of gate functionalization (*viz.* PDMS-printing *versus* immersion in the SAM solution) as well as the different morphology of the Au electrodes. In theory, it should be expected the *V*_th_ scales with the metal gate work function. However, not an obvious tendency whatsoever is found in this case. For instance, the long SAMs 1DT and PFDT should have an opposite effect on *V*_th_, but, surprisingly, they promote the same significant Δ*V*_th_, particularly in the EGOFET configuration where a gold disk is employed and the SAMs should be more packed. In fact, with the exception of the amino-terminated SAMs, the rest tend to shift positively the threshold voltage or to keep it close to the Au reference value.

Regarding the amino-terminated SAMs, the modification of the gate electrode through the PDMS stamp strongly impacts on the electrical transistor characteristic and a drastic decrease in *V*_th_ is observed. In the case of AUT, this effect could be probably ascribed to poor layer compactness which permits the protonation of the amino groups and increases the polarization of the gate surface. On the contrary, once this SAM is prepared on the disk electrode, a denser monolayer packing probably is expected to impede the protonation due to stronger electrostatic repulsions resulting in a lower Δ*V*_th_. The scenario is different for ATP; the short length of this molecule does not allow the formation of a compact monolayer and the effect on *V*_th_ remains comparable despite the employed SAM preparation technique.^43^

The plot of *I*_SD_*vs. V*_GS_ extracted from all the devices in the linear regime (*V*_SD_ = −0.1 V) undoubtedly reveals that, in addition to the *V*_th_ shift, the slope is also changing in all these devices. The slope in these plots is proportional to the product of the device mobility by the dielectric capacitance (*i.e.*, *μC*). From the initial electrical data recorded with the bare gate electrode, the device mobility was calculated for each device as previously reported, having always an average value of 0.03 (±0.02) cm^2^ V^−1^·s^−1^.^31^ Considering then that the mobility of the OSC keeps constant, we can estimate the device capacitance in the devices with the functionalized gate electrode. In the bottom panels of Fig. 4, the capacitance shifts with respect to the devices with bare gold are plotted for each SAM (Δ*C* = *C*_SAM_ − *C*_ref_). As observed, the SAMs cause a significant impact on the dielectric capacitance (*i.e.*, the electrical double layers). In fact, there is a decrease of the capacitance, with the exception of the printed fluorinated SAMs, FTP and PFDT, which could be ascribed to a lower molecular coverage permitting an enhanced diffusion of ions through the SAM. Noticeable though, the capacitance decrease is quite remarkable for the PFDT and 1DT SAMs on the gate gold disk EGOFETs, again indicating the high density of these SAMs.

The change in the dielectric capacitance of a device influences the *V*_th_. In a transistor with a lower capacitance dielectric, the application of a higher *V*_GS_ will be required to create the same electric field than the one achieved in a device with a larger dielectric capacitance. In the reported experiments, we believe that the tuning of the devices electrical properties and their *V*_th_ with the SAMs are mainly caused by how the SAMs affect the formation of the electrical double layers and, hence, how the total dielectric capacitance is modified. Most of the SAMs on the gate electrode lead to a decrease on the device capacitance, which concomitant increases the device *V*_th_. However, other factors such as the packing of the SAMs and their polarizability are also crucial in order to understand the final device response.

## Conclusions

The fabrication of a flexible Electrolyte-Gated Field-Effect Transistor (EGOFET) on Kapton® foil has been accomplished and we have successfully patterned a coplanar gate electrode and demonstrated its compliance for electrolyte-based measurements. Our characterization by means of water medium demonstrated the excellent electrical characteristic of the devices that are comparable with a standard top gate configuration. This compact architecture permits an optimal functionalization of the gate electrode through a printing technique, namely PDMS-assisted printing, which allowed the local tuning of the metal electrode properties without affecting the vicinity elements. Hence, a series of SAMs (*viz.* HS–R–X, where R is a saturated alkyl chain or an aromatic ring and X is an amino, carboxylic, fluorinated or a methyl group) have been casted onto the metal gate electrode as proof-of-concept. Thanks to our deposition technique, an efficient and fast functionalization have been achieved, as demonstrated by electrochemistry, Kelvin probe microscopy and contact angle measurements. SAMs printing on the gate electrode has revealed to be a smart option for modulating the EGOFET response. Our results show that the gate functionalization affects in a two-fold manner the device response, leading to potentiometric and capacitive effects. Although the latter effect seems to be the dominant one, disentangling these two contributions to provide a better control of the device properties still remains challenging.

## Conflicts of interest

There are no conflicts to declare.

## Supplementary Material

RA-008-C8RA05300F-s001
