# Supplementary material for: Modification of the gate electrode by self-assembled monolayers in flexible electrolyte-gated organic field effect transistors: work function vs. capacitance effects
Source: RSC Adv. 2018 Aug 2;8(48):27509–15. doi: 10.1039/c8ra05300f (PMC6333246; doi:10.1039/c8ra05300f)
Supplement: RA-008-C8RA05300F-s001 [file RA-008-C8RA05300F-s001.pdf]

Electronic Supplementary Material (ESI) for RSC Advances.
This journal is © The Royal Society of Chemistry 2018

Electronic Supporting Information (ESI)

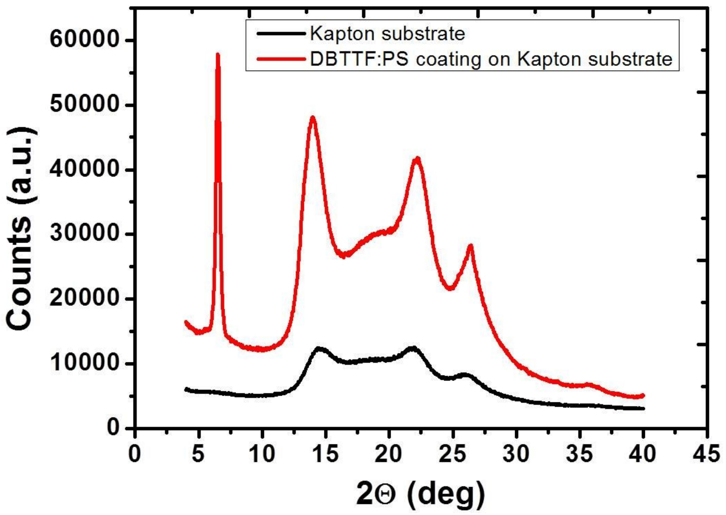

Figure S1 X-ray diffractogram of bare Kapton substrate and DBTTF:PS coated substrate. The peak at 6.55º is characteristic of the γ-phase of DBTTF.

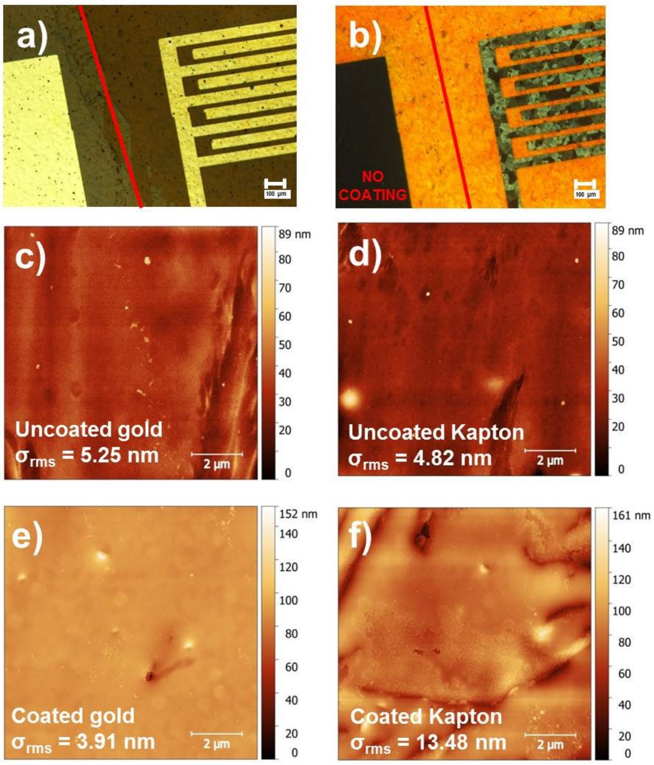

Figure S2 a) Optical microscopy image of S/D electrodes near the gate electrode region showing the patterned area of the semiconductor and b) its corresponding optical polarized image. c) and d) show the gold and Kapton® region of the devices and e) and f) the OSC blend-coated regions. It is worth mentioning that the scratch features are peculiar of Kapton® itself and they are not related to our process.

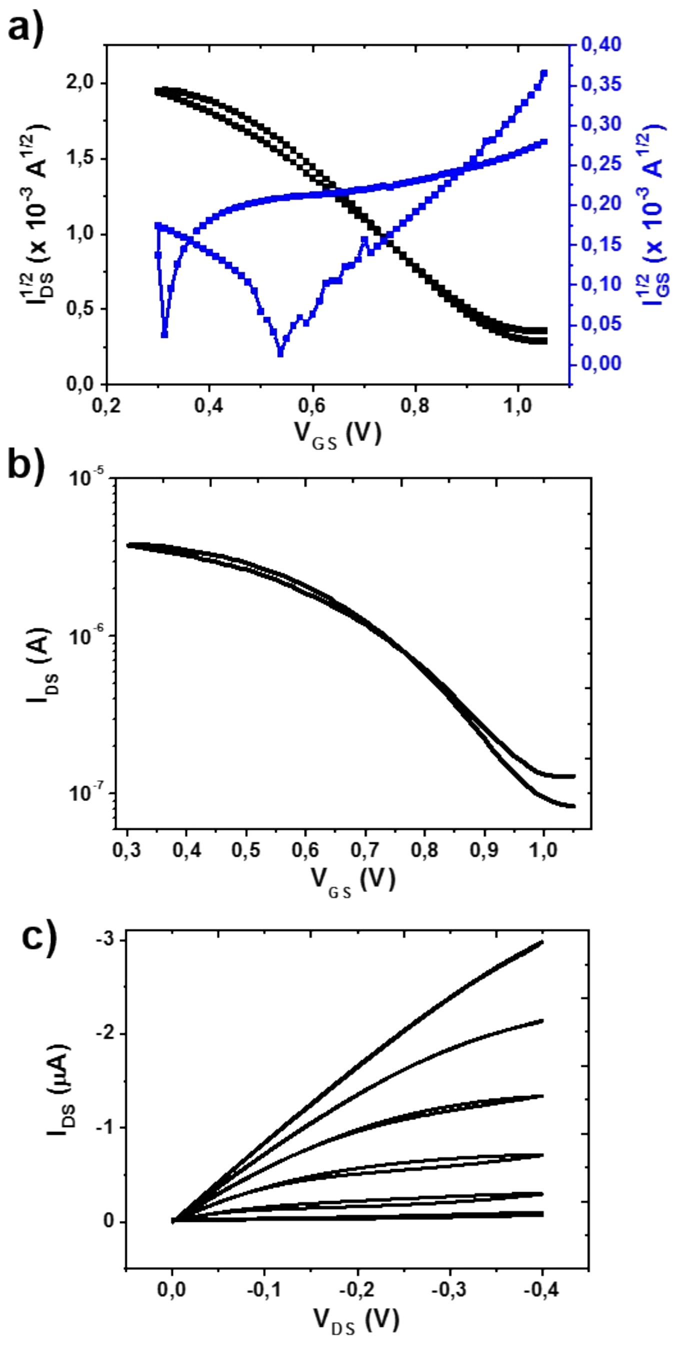

Figure S3 Lin-lin transfer characteristic and b) Log-lin transfer characteristic of EGOFET device recorded in saturation regime (V_{DS} = -0.4 V). c) Output characteristic of EGOFET device obtained by sweeping the gate voltage from 1 V to 0.5 V. All the measurements are performed in MilliQ water.

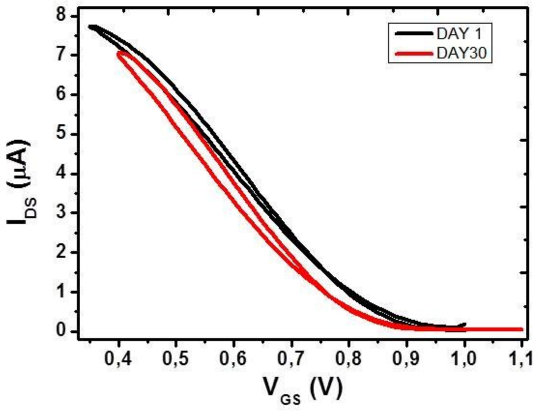

Figure S4 Transfer characteristics of DBTTF:PS flexible device measured with coplanar gate architecture at day 1 and after one month (V_{DS} = -0.1V).

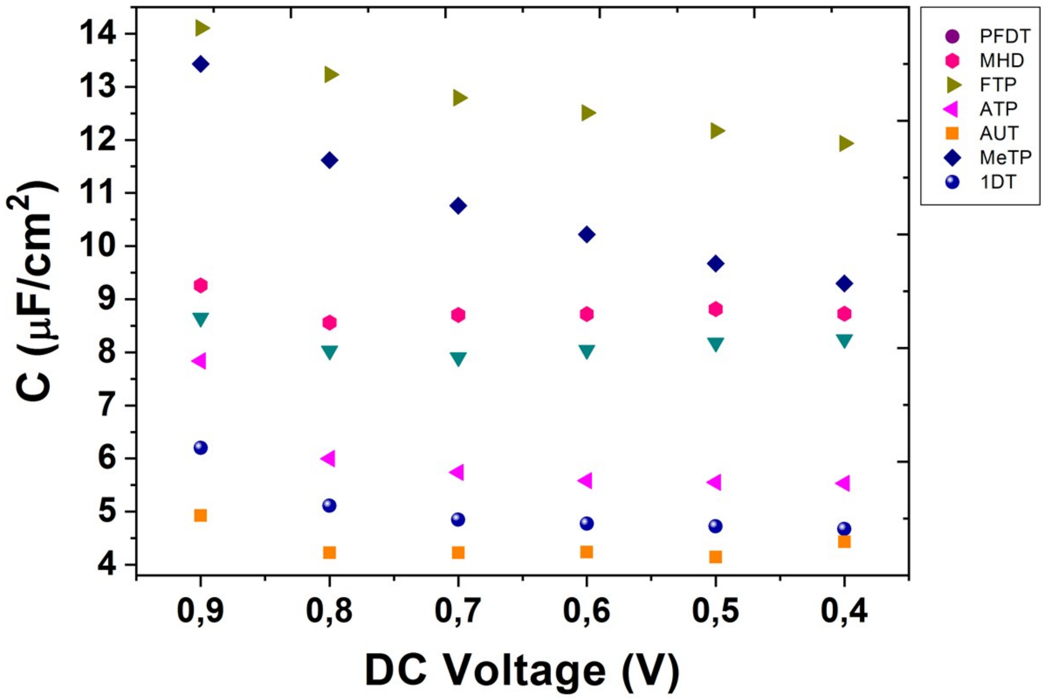

Figure S5 Capacitance behavior of each SAM recorded at different DC bias. The DC voltage range has been selected according to the operational gate voltage (V_{GS}) of the EGOFET device.

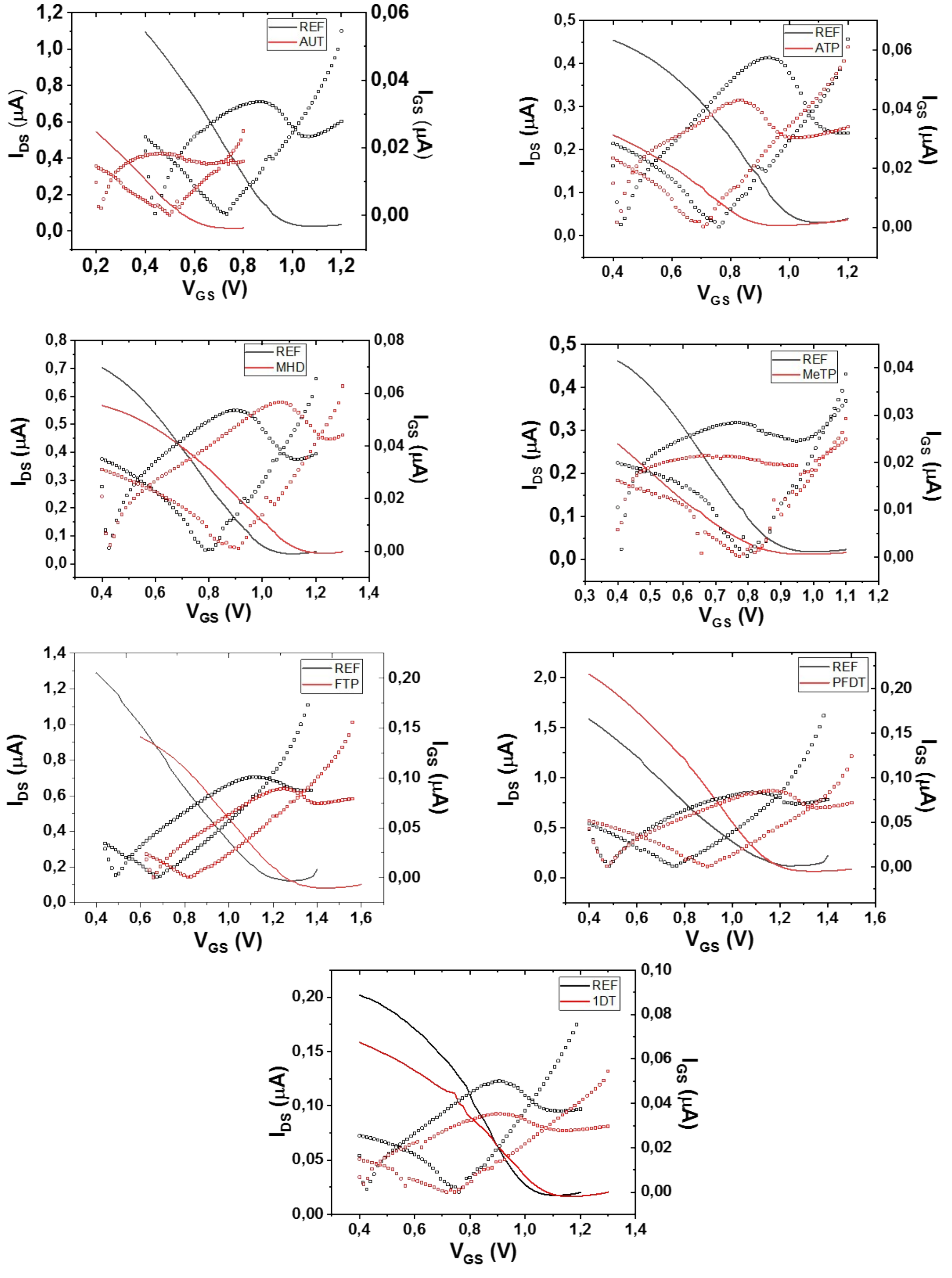

Figure S6 Transfer characteristic (linear regime V_{DS} = -0.1 V) measured for all devices with different SAM. Black curve refers to the reference measurement with a bare gate electrode while the red one has been recorded after the functionalization of the gate terminal with a SAM.
